# Endometrial disruption does not improve implantation in patients who have failed the transfer of euploid blastocysts

**DOI:** 10.1007/s10815-015-0435-0

**Published:** 2015-01-28

**Authors:** Marie D. Werner, Eric J. Forman, Kathleen H. Hong, Jason M. Franasiak, Paul A. Bergh, Richard T. Scott

**Affiliations:** 1 Robert Wood Johnson Medical School of Rutgers University, New Brunswick, NJ USA; 2 RMA of New Jersey, Basking Ridge, NJ USA; 3 140 Allen Road, Basking Ridge, NJ 07920 USA

**Keywords:** Endometrial Injury, Endometrial Scratch, Implantation, Pre-implantation genetic diagnosis, IVF

## Abstract

**Purpose:**

To assess the impact of single pass outpatient endometrial biopsy in patients at the highest risk for an endometrial cause for failed implantation; those that have failed to conceive despite the transfer of morphologically normal euploid embryos.

**Methods:**

This is a retrospective cohort study consisting of all patients less than 42 years old who failed their first euploid blastocyst transfer and subsequently completed a second transfer cycle of euploid blastocysts. Cycles were analyzed to determine if a single pass endometrial biopsy, termed 'endometrial disruption', was performed in a cycle preceding their second embryo transfer. Transfer outcomes were analyzed and implantation rates calculated. Data analysis was performed to compare outcomes between patients who had endometrial disruption performed versus those that did not.

**Results:**

Two hundred ninety patients failed their first euploid embryo transfer and subsequently completed a second euploid embryo transfer and were included. Thirty-nine patients underwent endometrial disruption and 251 did not. There were no statistical differences in clinical implantation rate or sustained implantation rate between the group with endometrial disruption and subjects without any intervention (Clinical IR, 43.6 % vs. 55.0 %, *p* = 0.13; 38.5 % vs. 42.6 %, *p* = 0.60). When controlling for transfer order there was no statistical difference noted in implantation rates.

**Conclusions:**

Single pass endometrial biopsy has no impact on endometrial receptivity in the highest risk subgroup- patient's that have failed to sustain the transfer of morphologically normal euploid embryos- as evidenced by equivalent implantation rates. It is possible that variations in technique may alter outcomes and randomized trials are needed to answer this question.

## Introduction

Implantation is a complicated biochemical process but fundamentally there are two key factors that are essential to successful implantation and establishment of a viable pregnancy: embryonic competence and endometrial receptivity. Over the past decade research has focused on embryonic factors and the selection of morphologically-superior, genetically-normal blastocysts for transfer [1, 2]. Now that substantial improvements in embryonic culture and enhanced selection techniques have been validated, there is a renewed focus on the potential reasons why high-quality, chromosomally-normal blastocysts do not always implant. If single embryo transfer pregnancy rates with advanced selection techniques are as high as 60 %, could the endometrium be culpable for up to 40 % of the failures [3]? Mechanical injury to the endometrium has been purported to enhance endometrial receptivity and improve embryo transfer outcomes, but research utilizing this technique has focused on either the general IVF population or those who have a history of failed implantation [4–9]. It is well known that most failed implantations are related to suboptimal embryo quality or aneuploidy. Even if endometrial disruption could enhance endometrial receptivity, it would not be able to improve the ability of non-viable embryos to implant. Patients who have previously had euploid blastocysts fail to implant would seemingly be at the highest risk of having an endometrial cause of failed implantation. This study seeks to address whether endometrial disruption improves outcomes in this high-risk population with prior failed euploid transfers.

The critical role of the endometrium during implantation is well characterized, but its role in implantation failure is poorly understood. A variety of factors (Matrix metalloproteinases (MMP), Interleukin-6 (IL-6), Leukemia inhibitory factor (LIF), Tumor necrosis factor alpha (TNF-α), Connexin 43 (Cx43), monocytes, macrophages) have been implicated as important, but an endometrial profile that promotes implantation has not yet been identified [10]. In the quest for the identification of a supportive endometrial profile, endometrial sampling prior to IVF has been studied. In one such analysis, the authors noted that 11 of 12 patients conceived after the endometrium was injured [11]. This unique finding led to subsequent studies evaluating the role of endometrial injury as a mechanism to enhance implantation. These studies unfortunately have yielded divergent results.

Mechanical endometrial injury in the cycle preceding IVF has been proposed to improve implantation. Local injury may induce decidualization or provoke wound healing, attracting cytokines, growth factors, LIF, and other immune modulators to the area to enhance implantation [12]. Alternatively, it may induce endometrial injury resulting in adhesions or inadequate proliferation in subsequent cycles thereby slowing endometrial development and potentially causing endometrial-embryonic dyssynchrony [13]. The investigation of endometrial disruption and its ultimate impact on outcome thus far has been inconclusive. A randomized controlled trial (RCT) of 134 patients with one or more failed IVF cycles demonstrated dramatic improvement in overall outcomes after endometrial disruption on repeated biopsies prior to transfer [5]. In a similar RCT, 100 patients with a history of failed IVF had endometrial sampling performed twice, which significantly improved the chance for live birth in the intervention group (22.4 % vs 9.8 % *P* = 0.04) [14]. However, a subsequent study utilizing a sham procedure showed no improvement [15]. In contrast, a large meta-analysis demonstrated that local endometrial injury is 71 % more likely to result in a clinical pregnancy as opposed to no intervention [4]. In the most recent literature, a retrospective oocyte donor model and a prospective trial with IVF/ICSI subjects both found no statistical difference between endometrial disruption and no intervention [16, 17].

This study seeks to assess the impact of single pass endometrial biopsy, termed endometrial disruption, in the sub-group of patients at highest risk for an endometrial cause for failed implantation: those who have failed to conceive after the transfer of morphologically-normal, euploid embryos. This population is difficult to manage and there are no clear guidelines to improve outcomes after a failed euploid transfer. To complete this analysis only those patients who failed to sustain implantation despite the transfer of morphologically-normal, euploid blastocysts were included. To determine whether endometrial disruption was beneficial, the outcomes in a subsequent transfer of euploid blastocysts were compared between the group who underwent endometrial disruption and those who did not.

## Materials and methods

### Study population

This was a retrospective analysis of all patients less than 42 years old who failed to deliver after their first euploid blastocyst transfer and subsequently completed a second transfer cycle of euploid blastocysts at the study center between 2010 and 2014. Both fresh and frozen transfer cycles were included. Patients were stratified into two groups: those who had endometrial disruption performed preceding their second embryo transfer (study group) and those who did not have this procedure performed (control group). Demographic information was collected and the most common diagnosis was male factor (21 %), with the distribution of diagnoses included in Table 1. ICSI was performed in all cases, as is necessary for CCS, possibly reducing the impact of male factor diagnoses recorded in this study. Five patients were categorized with uterine factor and in all cases the uterine anomaly was significant for fibroids that did not impact the endometrial cavity. The groups were similar in overall characteristics except that the group with no intervention was slightly older (33.9 ± 4.0 vs. 35.5 ± 4.0, *p* = 0.02). While this difference was statistically significant, it is not thought to be clinically relevant given that increasing age correlates with diminishing ovarian reserve and increasing aneuploidy prevalence, which were controlled for with the transfer of only euploid blastocysts. Additionally, patients had equivalent sonographic markers of endometrial development as measured by endometrial thickness (8.4 ± 1.6 mm vs. 8.8 ± 2.0 mm, *p* = 0.66) (Table 2).

**Table 1:** Distribution of diagnoses in patients with one failed euploid blastocyst transfer who subsequently completed a second transfer cycle of euploid blastocysts

Diagnosis	No intervention N (%)	Endometrial disruption N (%)	All patients N (%)
Combined male/Female	29 (12 %)	3 (8 %)	32 (11 %)
Diminished ovarian reserve	12 (5 %)	1 (3 %)	13 (4 %)
Endometriosis	10 (4 %)	2 (5 %)	12 (4 %)
Genetic	14 (6 %)	2 (5 %)	16 (6 %)
Male factor	54 (22 %)	8 (21 %)	62 (21 %)
Other factor	39 (16 %)	8 (21 %)	47 (16 %)
Ovulatory dysfunction	48 (19 %)	8 (21 %)	56 (19 %)
Tubal factor	16 (6 %)	1 (3 %)	17 (6 %)
Unknown factor	24 (10 %)	6 (15 %)	30 (10 %)
Uterine factor	5 (2 %)	0 (0 %)	5 (2 %)
Total patients	251	39	290

**Table 2:** Demographic information, cycle characteristics, and embryology outcomes in patients who failed their first euploid embryo transfer and subsequently completed a second euploid embryo transfer

Parameters	Endo Disruption (*N* = 39) (±SD)	No Intervention (*N* = 251) (±SD)	*p* value
Mean adjusted age (years)	33.9 ± 4.0	35.5 ± 4.0	0.02
Mean highest D3 FSH (IU/L)	8.1 ± 2.8	9.2 ± 6.7	0.66
Mean baseline antral follicle count	19.9 ± 12.1	16.9 ± 9.7	0.13
Mean endometrial thickness at transfer (mm)	8.4 ± 1.6	8.8 ± 2.0	0.66
Number of frozen cycles	38 (97.4 %)	237 (94.4 %)	0.43
Mean count of mature oocytes retrieved	12.8 ± 5.9	12.9 ± 7.1	0.63
Mean count of blastocysts biopsied	6.6 ± 4.4	5.7 ± 3.8	0.23
Mean count of euploid blastocysts transferred	1.5 ± 0.6	1.4 ± 0.5	0.16
Mean count of blastocysts cryopreserved	4.8 ± 3.4	4.3 ± 3.1	0.45

All stimulation and embryology techniques were performed as per routine practice standards. All embryos were cultured to the blastocyst stage of development regardless of size or quality of the cohort. All expanded blastocysts with a discernible inner cell mass underwent trophectoderm biopsy for Comprehensive Chromosome Screening (CCS) analysis on day 5 or 6 of embryo development. The morphologically-best, euploid embryo(s) were selected for transfer in all cycles. Fresh transfers were performed on the morning of day 6. Frozen transfers were performed in the afternoon of the sixth day of progesterone exposure, typically in the form of 50 mg intramuscular progesterone in oil. Fresh and frozen embryo transfer cycles were included as both techniques have equivalent pregnancy outcomes in the study center [3]. The decision on transfer order was based upon number and quality of euploid blastocysts available, demographic factors including patient age and prior history, patient preference, and guidance from the primary physician and clinical team. No more than two blastocysts were transferred in any cycle. Prior to retrieval and as per routine practice standards, uterine cavity evaluation was performed via saline-infusion sonogram (SIS) documenting no intrauterine pathology. This procedure is performed with a small flexible intrauterine insemination catheter (Rocket Duo; Rocket Medical, Hingham, MA) proceeding retrieval, and is only repeated in between transfer cycle one and two if the patient experienced a miscarriage of a clinically visible pregnancy on ultrasound.

### Technique of endometrial disruption

Due to the retrospective nature of the study design, a single pass endometrial biopsy, termed endometrial disruption was performed in cycles at the recommendation of the primary clinician. During the time period between 2010 and 2014 the technique of endometrial disruption was standardized at the study center and therefore comparable between clinicians and cycles. This procedure was performed via an endometrial cell sampler (Endocell; Wallach, Trumbull, CT) in the luteal phase of the menstrual cycle and was accomplished in the 1–2 cycles prior to embryo transfer. After introducing the catheter into the uterine cavity and withdrawing the piston, a single pass biopsy was performed. In this way, the procedure was low in cost, a minor intervention, and performed in the outpatient setting. Tissue collected was discarded and no further analysis was performed.

### Embryo transfer and follow-up

Embryo transfer was performed as per practice standards with an endometrial thickness of at least 6 mm documented in the transfer cycle of interest. Clinical implantation (clinical IR) was defined as the maximum number of gestational sacs per embryo transferred and sustained implantation (sustained IR) by the number of fetal heartbeats at discharge (approximately 8–9 weeks of gestation) per embryo transferred. A clinical miscarriage was defined as the loss of a pregnancy after visualization of a gestational sac.

### Statistical analysis

Data analysis was performed via Analyse-it for Excel version 2.30. Categorical data were analyzed using Chi square to compare outcomes between groups. Continuous variables are presented as a mean ± SD and were analyzed by Mann-Whitney U Tests. Statistical significance determined for *p* value <0.05. This retrospective analysis was IRB approved by Western IRB, protocol # 20021333.

## Results

In total, 290 patients who had failed their first euploid embryo transfer and subsequently completed a second euploid embryo transfer were identified and included for analysis. Each cycle was then analyzed to determine if endometrial disruption was performed in between transfer cycle 1 and cycle 2. Of all included patients, 39 (13 %) patients underwent endometrial disruption prior to their second euploid embryo transfer and 251 did not undergo this procedure. Of the 39 patients who underwent endometrial disruption, 32 (82 %) patients had the procedure performed within one menstrual cycle prior to transfer, and 7 (18 %) patients had the procedure performed within two menstrual cycles prior to transfer.

**Fig. 1:**
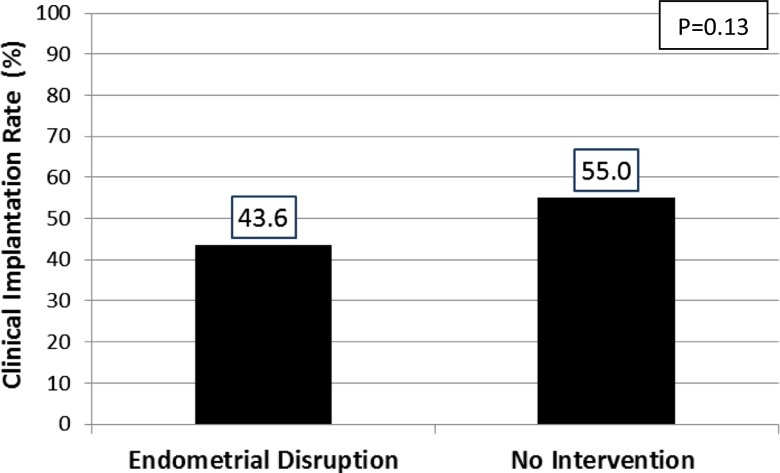
Clinical Implantation Rate is not significantly different between groups studied

A total of 403 euploid embryos were transferred, with an average of 1.4 embryos transferred per patient. There was no statistical difference between transfer order between groups; 51.3 % in the endometrial disruption group and 63.2 % in the control group were single embryo transfers (*N* = 20, *N* = 158; *p* = 0.16). Most included procedures were frozen embryo transfers, 97.4 % in the endometrial disruption group and 94.4 % in the control group, (*N* = 38, *N* = 237; *p* = 0.48). This can likely be attributed to the fact that supernumerary euploid embryos that were cryopreserved after a fresh cycle were transferred in the subsequent frozen cycle. The overall clinical implantation rate was 53.4 % and sustained implantation rate was 42.1 %. There were no statistical differences in clinical implantation between the group with endometrial disruption and the control group (43.6 % vs. 55.0 %, *p* = 0.13) (Fig. 1). Similarly, there was no difference between these groups in sustained implantation rates (38.5 % vs. 42.6 %, *p* = 0.60). The overall incidence of miscarriage was also not statistically different between groups (5.1 % vs. 12.4 %, *p* = 0.60). The mean age or transfer order did not differ between cycles resulting in a pregnancy and those that were unsuccessful. When fresh transfers were excluded, and only frozen cycles were analyzed, there were no statistical differences noted in clinical implantation, sustained implantation, or miscarriage rates between groups (42.1 % vs. 55.5 %, *p* = 0.06; 36.8 % vs. 43.2 %, *p* = 0.33; 5.3 % vs. 12.2 %, *p* = 0.15).

In the subgroup analysis, single and double embryo transfers were evaluated separately. In the investigation of single embryo transfers, no statistical differences was noted in clinical implantation rates between the group with endometrial disruption, and subjects without this procedure (45.0 % vs. 55.7 %, *p* = 0.38). Similarly no significant difference was noted in the analysis of double embryo transfers (42.1 % vs. 53.8 %, *p* = 0.20).

A power analysis was performed to reflect the statistical impact suggested by the aforementioned meta-analysis where endometrial injury performed in the cycle preceding ovarian stimulation was 70 % more likely to result in a clinical pregnancy [4]. This study had a 97 % power to detect the same impact in pregnancy rates, but found no such difference. Furthermore, this analysis included only high-quality, euploid blastocysts, thereby minimizing the variable of embryonic quality.

## Discussion

In the present retrospective study, endometrial disruption performed with a single pass endometrial biopsy did not improve pregnancy or sustained implantation rates in women who had previously failed the transfer of morphologically normal euploid blastocysts. The significant advantage of this study is the inclusion of only morphologically-normal, euploid blastocysts, thereby neutralizing many of the embryonic factors that could influence IVF outcomes. Identifying this high-risk sub-group for study is particularly impactful to clinical care as these are the patients who are offered investigational techniques with limited evidence to support them. Unfortunately this subgroup of patients still remains a challenging clinical dilemma as no improvement in outcome was seen with endometrial disruption.

Recent literature has demonstrated a potential improvement in clinical pregnancy and live birth rates with the use of endometrial disruption in the cycle proceeding IVF. It is plausible that the wound healing process may be provoked with endometrial injury and this can induce the release of immunomodulators that directly impact implantation [18]. Furthermore, this technique has been shown to upregulate genes important in implantation and endometrial receptivity [19]. Current literature is widely disparate on whether or not to recommend this treatment modality to patients, with conclusions ranging from marked improvement, no difference detected to a potentially-negative impact [4, 6, 16, 20]. Consequently the strength of this study is that it targets the subgroup of patients most likely to benefit from this treatment strategy, those who fail to deliver after the transfer of morphologically-normal euploid blastocysts.

For this same reason, the present analysis is not directly applicable to the general IVF population and this is a limitation of the study design. The theory of disrupting the endometrium in the cycle preceding transfer may still hold merit for other populations; however, in a recent prospective analysis targeting the general IVF population no such benefit was seen [17]. It is possible that a more aggressive approach to endometrial injury may be warranted in this population or that direct visualization with hysteroscopy may confer additional benefits as opposed to an outpatient single-pass biopsy. Further, it is possible that more than one cycle of endometrial injury may be necessary or the time span between biopsy and transfer may need to be adjusted. In this study endometrial disruption was only included if performed in the 1–2 cycles proceeding embryo transfer. In one analysis, benefit was found when endometrial disruption was as far removed as 6 cycles prior to transfer, however the optimal window of disruption has yet to be determined [21].

The limited sample size and the high incidence of frozen transfers may be confounding variables in this analysis. For this reason a separate analysis was performed documenting no difference in outcomes for frozen cycles. The high proportion of frozen transfers included in this study may have impacted outcomes as this may represent an exclusion of patients who did not have supernumerary euploid blastocysts available for transfer in a subsequent cycle. Additionally, it may have been useful to analyze the biopsy results for histopathologic diagnosis, potentially providing more information to direct cycle management. As a result of the retrospective nature of this study design, we cannot exclude the possibility that women who underwent endometrial disruption could have been an even poorer prognosis group then those who did not and subsequently underwent this procedure at the recommendations of the clinical team. However, it is reassuring to note that both populations had equivalent demographic parameters and cycle dynamics, even with the potential for a slighter poorer prognosis in the intervention group, given the older age of these patients. All patients in this study had a saline infusion sonogram to document a normal intrauterine cavity prior to embryo transfer and while there has not been evidence suggesting this procedure alters the endometrial environment, it could theoretically be a confounding variable to study design.

Implantation failure is a challenging clinical dilemma and both the embryo and endometrium play critical roles in establishing a successful pregnancy. Recent advances in blastocyst culture and genetic screening have allowed for selection of the highest-quality embryos; however, tools that can enhance endometrial receptivity are still under investigation. This is the first analysis that addresses the impact of endometrial disruption in the highest-risk subgroup: those who have previously failed the transfer of morphologically–normal, euploid blastocysts.

Single pass endometrial biopsy has no measurable impact on endometrial receptivity in the highest risk subgroup -patients who have failed to sustain the transfer of morphologically normal euploid embryos- as evidenced by equivalent clinical and sustained implantation rates. While it remains possible that the etiology of the failures may relate to embryo quality in spite of rigorous efforts to identify morphologically-normal, euploid embryos, these data suggest that caution should be used prior to adopting endometrial disruption and that class I data evaluating specific well-defined clinical situations are still needed. Specifically, a randomized controlled trial evaluating live birth outcomes following endometrial disruption in the cycle immediately preceding fresh or frozen euploid blastocyst transfers is needed to determine the clinical efficacy of this strategy in patients who fail to sustain implantation of the highest-quality embryos.
